# La choroïdite serpigineuse like et tuberculose intraoculaire: à propos de 2 cas et revue de la littérature

**DOI:** 10.11604/pamj.2015.20.367.3859

**Published:** 2015-04-15

**Authors:** Hakima Elouarradi, Samira Tachfouti, Lalla Ouafae Cherkaoui, Othmane Charhi, UChama Daoudi, Saloua Khalil, Nouha Zerkaoui, Amal Alouane, Kamal Naciri, Rajae Daoudi

**Affiliations:** 1 Université Mohammed V Souissi, Service d'Ophtalmologie A de l'Hôpital des Spécialités, Centre Hospitalier Universitaire, Rabat, Maroc

**Keywords:** Tuberculose, choroïdite serpigineuse like, traitement antituberculeux, Tuberculosis, Serpiginous-like choroiditis, TB treatment

## Abstract

Les manifestations oculaires de la tuberculose sont très polymorphes. La choroïdite serpigineuse like représente une forme clinique rare de la maladie. Les auteurs rapportent l'observation de deux patients issus d'un pays d'endémie tuberculeuse, présentant un tableau de choroïdite serpigineuse like avec un bilan étiologique orientant le diagnostic vers une tuberculose oculaire. Un traitement antituberculeux a été préconisé en association avec une corticothérapie, l’évolution était marquée par une stabilisation des lésions et une discrète amélioration de l'acuité visuelle. Dans les pays d'endémie tuberculeuse le diagnostic doit être évoqué en présence d une choroïdite multifocale serpigineuse like afin de proposer un traitement adapté.

## Introduction

Les manifestations intra oculaires de la tuberculose peuvent être très variées, comprenant les choroïdites, les uvéites antérieures ou intermédiaires, souvent granulomateuses, les vascularites rétiniennes, la choroïdite serpigineuse like, la pan uvéite, et l'endophtalmie [1]. Aucune de ces manifestations cliniques n'est pathognomique. Certes, le diagnostic de certitude de la tuberculose intraoculaire repose sur la présence de mycobactéries (ou de leur génome) au niveau des prélèvements oculaires, ce qui est de réalisation très difficile en pratique clinique quotidienne. Le diagnostic serait donc dans la majorité des cas présomptif, posé devant un ensemble d’éléments d'orientation cliniques et para cliniques. Parmi les manifestations intraoculaires de la tuberculose, la choroïdite serpigineuse like représente une forme clinique rare de la maladie, bien que cette association a été reconnue depuis des décennies. Nous rapportons deux observations de choroïdite serpigineuse d origine tuberculeuse.

## Patient et observation

### Première observation

**Figure 1:**
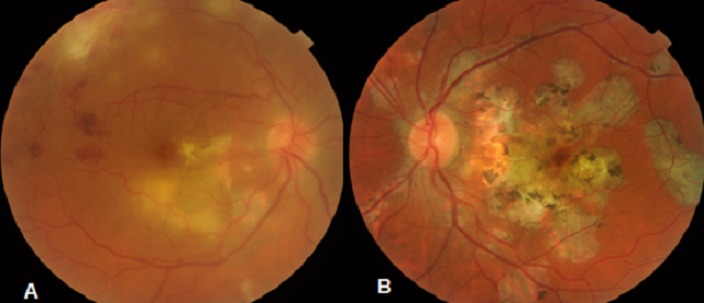
Aspect du Fond d’œil: (A) les lésions au niveau de l’œil droit sont actives jaunâtres à bords flous avec une vascularite rétinienne en regard et de multiples hémorragies en taches; (B) les lésions au niveau de l’œil gauche sont cicatricielles grisâtres à contours nets

**Figure 2:**
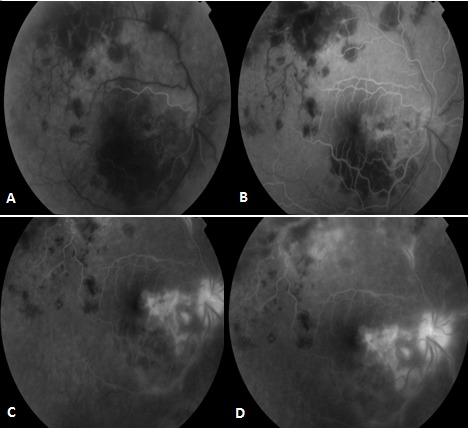
Aspect à l'angiographie à la fluorescéine de l’œil droit: (A) et (B) hypo fluorescence au centre des lésions choroïdiennes aux temps précoces; (C) et (D) hyper fluorescence des bords aux temps tardifs

**Figure 3:**
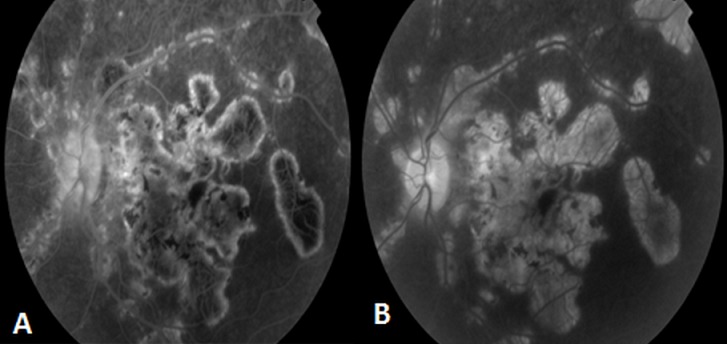
Aspect à l'angiographie à la fluorescéine de l’œil gauche (A) Les lésions sont hypo fluorescentes au centre avec hyper fluorescence des bords d'emblée dès les temps précoces; (B) hyper fluorescence centrale progressive aux temps tardifs

**Figure 4:**
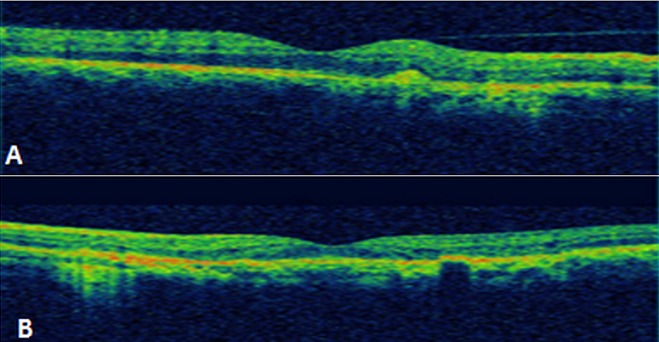
OCT maculaire: (A) à droite un œdème maculaire central avec hyper réflectivité de la choriocapillaire en rapport avec les foyers de choroïdite (B) à gauche un épaississement diffus de l’épithélium pigmentaire sans œdème maculaire

Patient de sexe masculin âgé de 57 ans, bien vacciné, tabagique chronique, ayant comme antécédent un père traité pour tuberculose pulmonaire, et qui a présenté 3 mois auparavant des épisodes de rougeur, douleur oculaire et une baisse de l'acuité visuelle de l’œil gauche bilatéralisée 2 mois plutard. L'acuité visuelle corrigée est à 8/10 au niveau de l’œil droit (OD) et 7/10 au niveau de l’œil gauche(OG), le tonus est à 14mmHg en ODG. L'examen bio microscopique trouve un segment antérieur calme en ODG. Le fond d’œil révèle au niveau des 2 yeux un tyndall vitréen cellulaire modéré, ainsi que la présence de foyers de choroïdite bilatérale à répartition géographique, situés en péri papillaire, évoluant de façon centrifuge épargnant le centre de la macula. Ces lésions sont actives jaunâtres à bords flous avec une vascularite rétinienne en regard et de multiples hémorragies en taches au niveau de l’œil droit (Figure 1, A), et cicatricielles grisâtres à contours nets au niveau de l’œil gauche (Figure 1, B). L'angiographie à la fluorescéine montre une hypo fluorescence au centre des lésions choroïdiennes avec une hyper fluorescence des bords aux temps tardifs à droite (Figure 2), à gauche Les lésions sont hypo fluorescentes au centre avec hyper fluorescence d'emblée dès les temps précoces (Figure 3, A) puis hyper fluorescence centrale progressive aux temps tardifs (Figure 3, B). L'OCT maculaire a objectivé à droite un œdème maculaire central avec hyper réflectivité de la choriocapillaire en rapport avec les foyers de choroïdite (Figure 4, A), avec à gauche un épaississement diffus de l’épithélium pigmentaire sans œdème maculaire (Figure 4, B). La numération formule sanguine, le bilan inflammatoire (vitesse de sédimentation et CRP), et la radiographie thoracique sont normaux. L'intradermoréaction à la tuberculine est phlycténulaire à 15mm, le dosage du quantiferon est positif, le reste du bilan étiologique est normal. Le diagnostic de tuberculose intraoculaire a été retenu. Un traitement antituberculeux a été préconisé associant rifampicine 10 mg/ kg / jour, isoniazide 5 mg/ kg / jour, éthambutol 15 mg / kg / jour et pyrazinamide 20 mg / kg/jour associe a une corticothérapie instaure 48 heures plutard sous forme d'un bolus de méthylprédnisolone à raison de 1g/j pendant 3 jours relayé par la prednisone par voie orale à raison de 1 mg/kg/j. L’évolution était marquée par la stabilisation des lésions, l'acuité visuelle corrigée est à 8/10 au niveau des 2 yeux au dernier contrôle.

### Deuxième observation

Patiente âgée de 37 ans, Antécédent de mère et de grand père traités pour tuberculose pulmonaire, qui consulte pour douleur, rougeur et baisse de l'acuité visuelle droite d'installation rapidement progressive depuis vingt jours.

**Figure 5:**
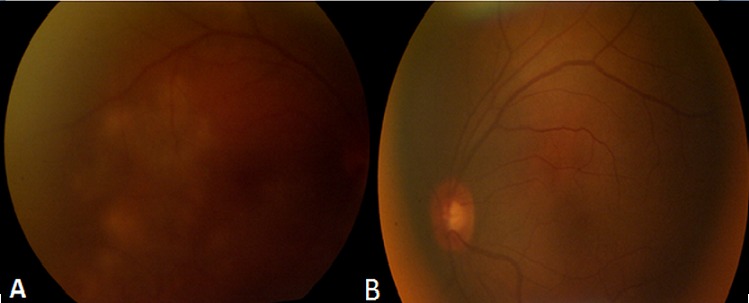
(A) Aspect de lésions de choroïdite serpigineuse au niveau de l’œil droit actives jaunâtres à bords flous avec un œdème papillaire, maculaire et vascularité rétinienne; (B) L’œil gauche est normal

**Figure 6:**
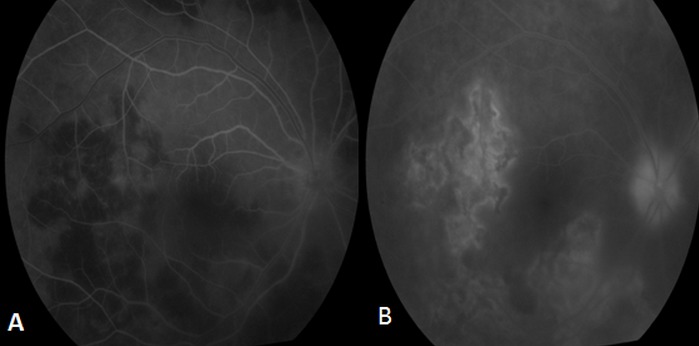
Aspect angiographique de l’œil droit (A) Temps précoce: hypo fluorescence au centre des lésions choroïdiennes; (B) Temps tardif: hyper fluorescence progressive des bords

**Figure 7:**
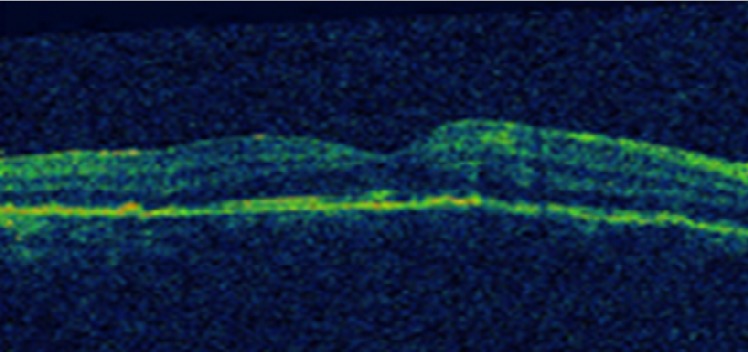
OCT maculaire: présence d’œdème maculaire central avec hyper réflectivité des couches externes de la rétine en rapport avec les foyers de choroïdite

L'examen clinique a trouvé une acuité visuelle corrigée à 6/10 au niveau de l’œil droit (OD) et à 9/10 au niveau de l’œil gauche, le tonus est normal à 12mmHg en ODG. L'examen bio microscopique trouve un segment antérieur calme au niveau des 2 yeux. Le fond d’œil révèle à droite un tyndall vitréen cellulaire avec présence de lésions de choroïdite serpigineuse like, avec des foyers choroïdiens en carte géographique, irréguliers, situés en péri papillaire, évoluant de façon polypoidale centrifuge. Ces lésions de l’œil droit sont actives jaunâtres à bords flous avec un œdème papillaire, maculaire et vascularite rétinienne (Figure 5, A). L'examen clinique de l’œil gauche est normal (Figure 5, B). L'angiographie à la fluorescéine montre une hypo fluorescence au centre des lésions choroïdiennes (Figure 6, A)avec une hyper fluorescence des bords aux temps tardifs (Figure 6, B). L'OCT maculaire a confirmé la présence de l’œdème maculaire central (Figure 7). La numération formule sanguine, le bilan inflammatoire (vitesse de sédimentation et CRP), et la radiographie thoracique sont normaux. L'intradermoréaction à la tuberculine est phlycténulaire à 16mm, le dosage du quantiferon est positif, le reste du bilan infectieux est normal. Le diagnostic de tuberculose intraoculaire est posé. Un traitement antituberculeux (rifampicine 10 mg/ kg / jour, isoniazide 5 mg/ kg / jour, éthambutol 15 mg / kg / jour, pyrazinamide 20 mg / kg/jour) instauré puis association d'un bolus de méthylprédnisolone pendant 3 jours relayé par une corticothérapie par voie orale à raison de 1 mg/kg/j. L’évolution était marquée par la stabilisation des lésions et une discrète amélioration de l'acuité visuelle à 7/10 corrigé au niveau de l’œil gauche.

## Discussion

Si les tubercules choroïdiens, dit de Bouchut, sont la manifestation intraoculaire la plus fréquente d'une tuberculose systémique [1], les uvéites tuberculeuses peuvent se présenter sous la forme d'une choroïdite serpigineuse-like ou encore appelée choroïdite serpigineuse multifocale d'origine tuberculeuse [2] ayant un aspect clinique similaire à la choroïdite serpigineuse qui elle, dite idiopathique, d’étiologie indéterminée. Il s'agit d'une affection inflammatoire rare, multifocale, chronique et récidivante ou Les lésions intéressent l’épithélium pigmentaire rétinien, la choriocapillaire et la choroïde, en s’étendant typiquement de la région juxta papillaire de façon polypoidale centrifuge, avec des dommages irréversibles des photorécepteurs [1]. Des formes à début maculaire ou évoluant à partir d'une AMPPE (Acute Multifocal Placoide Pigment Epitheliopathy) ont également été décrites. Les deux yeux sont souvent touchés de manière asymétrique. Il n'y a généralement pas de réaction inflammatoire du segment antérieur, mais une très discrète hyalite peut être présente. Chez nos deux patients, Les lésions choroïdiennes confluentes ont progressé de manière centrifuge à contours géographiques à partir de la papille, sans réaction inflammatoire significative du segment antérieur, ni du vitré. L'atteinte par contre était unilatérale chez l'un des deux patients, bilatérale chez l'autre. La pathogénie de l'affection reste un sujet de controverse [3] bien que de nombreuses hypothèses aient été émises. En fait, l’œil peut être la voie d'entrée de mycobactéries (tuberculose oculaire primaire) ce qui est très rare. Dans la plus grande majorité des cas, les bactéries atteignent l'intérieur de l’œil par dissémination hématogène (tuberculose oculaire secondaire). Une étude récente, ayant montré la multiplication de mycobactéries au sein de l’épithélium pigmentaire rétinien qui servirait de réservoir pour l agent pathogène [4].

Par ailleurs, plusieurs auteurs considèrent que la réaction inflammatoire secondaire à l'infection tuberculeuse est due essentiellement à une réaction d'hypersensibilité contre les antigènes mycobactériens. L'angiographie à la fluorescéine et au vert d'indocyanine peuvent être utile dans l’évaluation de l’étendue et de l'activité des lésions. L'analyse des clichés aux temps précoces montre une hypo fluorescence des lésions actives, suivie d'une hyper fluorescence tardive de façon centripète. Les lésions cicatricielles peuvent être hypo fluorescentes. Les clichés aux temps précoces et moyens montrent un liseré continu hyper fluorescent qui borde l'ensemble du territoire cicatriciel [5]. L'angiographie au vert d'indocyanine est plus précise que l'angiographie rétinienne à la fluorescéine dans l’évaluation de l’étendue des lésions. Les lésions se caractérisent par des zones d'hypo fluorescence sur les temps précoces et les temps tardifs, qui peuvent être étendues au delà des lésions observables en angiographie à la fluorescéine et en auto fluorescence. [5]

La tomographie en cohérence optique détermine très précocement l'extension des lésions, et objective la présence ou non d’œdème maculaire. L'association entre la choroïdite serpigineuse et la tuberculose a été rapportée il y'a plus d une dizaine d'années par Laatikainen L et Erkkila H [6]. En 2003, Gupta V et collaborateurs ont décrit sept cas de tuberculose oculaire présumée prenant l'aspect de choroïdite serpigineuse, avec une amélioration clinique sous l'association de traitement antituberculeux et corticothérapie [7]. De même, Mackensen et collaborateurs ont montré que 52% de leurs patients présentant une choroïdite serpigineuse avaient un test au Quantiféron positif et que 25% des patients s'amélioraient sous traitement antituberculeux associé à une corticothérapie [8].

Plus récemment, Nazari Khanamiri et Narsing A. Rao ont défini la choroïdite serpigineuse like chez les patients avec tuberculose oculaire présumée sous le nom de choroïdite serpigineuse multifocale d'origine tuberculeuse [2]. Le diagnostic de tuberculose oculaire repose souvent sur la mise en évidence d'une infection systémique latente ou active chez un patient en région d'endémie ou ayant été en contact avec des sujets tuberculeux. La radiographie thoracique peut montrer des lésions parenchymateuses médiastinales et pleurales qu'on pourrait mieux analyser sur le CT- scanner et le PET scan, mais elle peut être normale chez 15% des patients ayant une tuberculose présumée. De même, chez les patients présentant une tuberculose latente, la radiographie thoracique est souvent normale, ou peut montrer des lésions suggestives d'anciennes tuberculoses (nodules pulmonaires, cicatrices fibrosées, bronchiectasies..). Plus rarement, la mise en évidence du génome de Mycobactérium tuberculosis à partir de prélèvements oculaires, de même que la réponse clinique favorable aux traitements antituberculeux représente également une preuve diagnostic de BK oculaire.

En fait, la PCR, dont la sensibilité reste modérée, avec un grand risque de faux négatifs, est la seule technique qui permet à l'heure actuelle d'apporter des preuves directes de l'origine tuberculeuse d'une uvéite à partir des prélèvements. Malheureusement, elle n'est disponible que dans certains centres.

Chez nos patients, le diagnostic de tuberculose oculaire était posé sur un faisceau d arguments cliniques et para cliniques notamment la notion de contage tuberculeux, l'intradermoréaction à la tuberculine positive phlycténulaire, le test au quantiferon positif et l’évolution favorable sous traitement antituberculeux.

Le traitement d'uvéite tuberculeuse est basé sur l'association antituberculeux et corticoïdes. Chez nos malades, on a proposé une quadrithérapie de 2 mois: Isoniaside 5m/kg/j, Rifampicine 10mg/kg/j, Pyrazinamide 20mg/kg/j, Ethambutol 15mg/kg/j, suivie d'une bithérapie: Isoniaside et Rifampicine à la même posologie pendant quatre mois, associe au bolus de corticothérapie de trois jours par voie intraveineuse suivie d'une corticothérapie par voie orale pendant six semaines.

Le pronostic des choroïdites serpigineuses like tuberculeuses est favorable sous traitement anti bacillaire associé à une corticothérapie qui réduit considérablement les récidives oculaires avec une stabilisation voire une amélioration de l acuité visuelle finale en l'absence d'atteinte maculaire [9].

## Conclusion

La choroïdite serpigineuse like est une manifestation rare de la tuberculose intraoculaire, dont la confirmation diagnostique reste un défi dans la plupart des cas. Elle survient le plus souvent dans le cadre d'une tuberculose présumée ou latente dont la difficulté réside dans la mise en évidence de mycobactéries en intraoculaire Le diagnostic doit être évoqué devant toute choroïdite serpigineuse like notamment en pays d'endémie tuberculeuse afin de proposer un traitement adapté permettant une stabilisation ou une amélioration visuelle.
